# Spinal Epidural Cavernous Hemangiomas: A Clinical Series of 9 Cases and Literature Review

**DOI:** 10.3389/fonc.2021.572313

**Published:** 2021-03-17

**Authors:** Liyan Zhao, Yining Jiang, Yubo Wang, Yang Bai, Ying Sun, Yunqian Li

**Affiliations:** ^1^Department of Clinical Laboratory, Second Hospital of Jilin University, Changchun, China; ^2^Department of Neurosurgery, First Hospital of Jilin University, Changchun, China

**Keywords:** cavernous hemangiomas, spinal epidural lesions, epidural, diagnosis, treatment, prognosis

## Abstract

**Objective:**

Spinal epidural cavernous hemangiomas are very rare vascular lesions and are, therefore, seldom reported and easily misdiagnosed. Herein, we present a series of 9 cases with spinal epidural cavernous hemangiomas and discuss their pathogenesis, clinical characteristics, radiological findings, differential diagnosis, surgical interventions, pathological characteristics, and prognosis.

**Methods:**

We retrospectively retrieved and analyzed the data of patients with pure epidural cavernous hemangiomas, who underwent spinal magnetic resonance imaging, and surgical intervention at the First Hospital of Jilin University, China, between January 2005 and December 2019. The data on patients’ clinical manifestations, imaging characteristics, surgical intervention, histopathological findings, and postoperative follow-up were also recorded and analyzed.

**Results:**

In all, 5 men and 4 women with the mean age of 61 years (range, 41–78 years) were recruited. All patients experienced a gradual onset of symptoms and a slowly progressive clinical course, and no patient presented an acute onset of symptoms. The clinical manifestations include myelopathic signs in 8 patients (88.9%) and radicular symptoms in 3 patients (33.3%). On T1-weighted imaging, 6 lesions appeared isointense (55.6%), and 4 lesions exhibited hypointense (44.4%) signals. On T2-weighted imaging, 8 lesions appeared hyperintense (88.9%), and 1 lesion was heterogeneously intense (11.1%). Following gadolinium administration, 5 lesions appeared homogeneous with significant enhancement (55.6%), 1 lesion was homogeneous and mild enhancement (11.1%), and 3 lesions were heterogeneous with mild enhancement (33.3%). All patients received early microsurgery assisted by intraoperative electrophysiologic monitoring and neuronavigation in the lateral position *via* the posterior midline approach. Five patients underwent total laminectomy (55.6%), and 4 underwent hemilaminectomy (44.4%). Total excision was achieved in all cases. The average follow-up period was 55.1 months (ranging 10–123 months). All patients exhibited significant clinical improvement of their neurological deficits and achieved a favorable outcome with no recorded recurrence at last follow-up.

**Conclusions:**

Spinal epidural cavernous hemangiomas are rare vascular malformations. Early surgical treatment with total resection is an optimum treatment, particularly for patients with an acute exacerbation onset. The prognosis is mostly good and depends predominantly on the severity of the preoperative status.

## Introduction

Epidural cavernous hemangiomas (ECHs) are rare benign vascular malformations that can develop in any part of the central nervous system (CNS) (1, 2), and are predominantly observed in the cerebral hemisphere, cerebellum, and brainstem (2, 3). Their clinical manifestations are frequently determined by their localization. Historically, numerous nomenclatures have been used to describe these vascular malformations, including cavernous hemangioma, cavernous malformations, and venous malformations, based on the pioneering work on a classification scheme by Mulliken and Glowek in 1982 (2, 4, 5). However, there is considerable discordance among neurosurgeons because of the inaccurate and inconsistent use of various terminologies both in medical literature and in practice (6). In the spine, ECHs constitute of approximately 4% of all epidural space-occupying lesions and 5%–12% of all spinal vascular malformations (7–9). Notably, the lesions arise most frequently in the vertebral corpus with or without extension into the epidural space, followed by intramedullary localization, or the intradural extramedullary space; however, they are rarely purely localized in the extradural space (9, 10). The recent advent of neuroradiologic imaging techniques has allowed a significant increase in the number of cavernoma diagnoses. However, thus far, only approximately 90 cases have been reported in the literature (11–13). Herein, we present a series of 9 cases with pathologically confirmed pure spinal ECHs in patients who underwent surgical intervention at the First Hospital of Jilin University between 2005 and 2019 and discuss their clinical characteristics, radiological findings, differential diagnosis, treatment opportunities, and spinal ECH prognosis.

## Materials and Methods

This study was approved by the Ethics committee of the First Hospital of Jilin University, China. We retrospectively retrieved and analyzed the data of patients with pure ECHs, who received histopathological confirmation and underwent spinal magnetic resonance imaging (MRI) and surgical intervention at the First Hospital of Jilin University between January 2005 and December 2019. A total of 9 patients with pure ECHs were identified and included in this study. The clinical manifestations, neurological examinations, radiographic profiles, surgical interventions, pathologic examinations, and prognosis were recorded. The neurological status of the patients was assessed with the Frankel grading system as follows: complete neurological deficit (Grade A); sensory function only below the injury level (Grade B); incomplete motor function below the injury level (Grade C); fair to good motor function below the injury level (Grade D); free of neurological deficit or complete recovery (Grade E). The clinical assessment comprised of a physical examination, laboratory investigations, and MRI performed pre- and postoperatively and during routine postoperative follow-up visits by the attending neurosurgeon and resident neurosurgeon.

MRI was performed with the phased array spine coil and 1.5T superconducting magnet (Magnetom 3T, Siemens Healthcare, Erlangen, Germany). Pre-contrast fast spin-echo (FSE) T1-weighted imaging (T1WI; TR, 250–700 ms; TE, 12–30 ms), T2-weighted imaging (T2WI), and fat-saturated T2WI (TR, 2000–5000 ms; TE, 80–120 ms) and post-contrast T1WI in the transverse and sagittal planes were acquired in all cases. Post-contrast T1WI images were obtained after intravenous administration of gadopentetate dimeglumine (0.1 mmol/kg, Magnevist; Schering, Berlin, Germany) in all cases. The MRI findings were analyzed and summarized based on the assessments by an experienced radiologist at the First Hospital of Jilin University.

All patients underwent microsurgery assisted by neuronavigation and intraoperative electrophysiologic monitoring of body and limb sensation and motion. The extent of resection was classified based on postoperative imaging findings as gross total resection (GTR, complete removal of the lesion) and subtotal resection (STR, partial or incomplete removal). To confirm the diagnosis, histopathological examinations were performed on all specimens. Postoperatively, all patients included in this study were followed up with routine clinical and radiologic assessments.

## Results

### Patient Demographics and Clinical Characteristics

The demographic characteristics of the 9 included patients (5 men and 4 women) are summarized in **Table 1**. The mean age of the patients (± standard deviation) was 61 ± 12.2 years (range, 41 to 78 years). The interval between initial diagnosis and hospitalization ranged from 10 days to 4 years (average, 10.7 months). All patients experienced a gradual onset of symptoms and showed a slowly progressive clinical course; no patient experienced an acute onset of symptoms. The clinical manifestations included limb motor deficits causing poor muscle power and weakness in 5 patients (55.6%), sphincter disturbances resulting in urinary dysfunction and abdominal distension in 2 patients (22.2%), and sensory dysfunction triggering paresthesia and/or hypoesthesia in 7 patients (77.8%). Myelopathic signs were noted in 8 patients (88.9%), and radicular symptoms were recorded in 3 patients (33.3%).

**Table 1:** Clinical data of 9 patients with pure spinal epidural cavernous hemangiomas.

Case No.	Age(y)/ sex	Symptoms duration	Main symptoms and signs	Initial symptoms	Admission symptoms	Myelopathy	Radiculopathy	Preoperatie diagnosis	Emergency surgery	Surgical approach	Duration of operation	Intraoperative blood loss (mL)	Extent of resection
1	52/M	6 months	Numbness below 5 cm above the umbilical plane combined with acraturesis for 6 months and aggravated for 2 days. The myodynamia of lower limbs was of IV Grade.	Myelopathy	Myelopathy	Y	None	Cavernous hemangioma	No	Lateral position; Posterior midline approach; Total laminectomy	3h10min	300	GTR
2	73/M	2 months	Assessed by the health check and the patient presented no discomfort.	None	None	None	None	Schwannoma	No	Lateral position; Posterior midline approach; Hemi-laminectomy	4h57min	100	GTR
3	70/F	1 month	Progressive aggravate pain of back and lower limbs. Both lower limbs myodynamia were III Grade.	Radiculopathy	Radiculopathy	Y	Y	Schwannoma	No	Lateral position; Posterior midline approach; Total laminectomy	1h50min	50	GTR
4	41/F	20 days	Numbness below 2 cm above the umbilical plane. The myodynamia of lower limbs were of IV Grade.	Myelopathy	Myelopathy	Y	None	Cavernous hemangioma	No	Lateral position; Posterior midline approach; Hemi-laminectomy	3h25min	350	GTR
5	63/F	4 years	Numbness in the left leg for 4 years and symptoms aggravated in 2 years. She experienced numbness in the lower limbs, pain, and weakness. The myodynamia of lower limbs were of III Grade.	Myelopathy	Myelopathy	Y	Y	Meningioma	No	Lateral position; Posterior midline approach; Hemi-laminectomy	3h40min	300	GTR
6	60/F	10 days	Double upper limbs numbness and sensory disturbances. The myodynamia of four limbs were of IV Grade.	Myelopathy	Myelopathy	Y	None	Cavernous hemangioma	No	Lateral position; Posterior midline approach; Hemi-laminectomy	3h30min	250	GTR
7	47/M	1.5 month	He experienced numbness in the right lower limb and had poor activities.	Myelopathy	Myelopathy	Y	None	Schwannoma	No	Lateral position; Posterior midline approach; Total laminectomy	2h7min	1000	GTR
8	78/M	1 month	History of falls in 1 month before hospitalization, and the patient had abdominal distension and the right lower limb had sensory disturbance. The double lower limbs were weak, and the myodynamia were of II Grade.	Myelopathy	Myelopathy	Y	None	Schwannoma	No	Lateral position; Posterior midline approach; Total laminectomy	2h35min	200	GTR
9	61/M	3 years	He experienced back pain and numbess in lower limbs for 3 years. And both lower limbs weakened in 2 months before the hospitalization. Double lower limbs myodynamia of IV Grade; the disappearance of tendon reflex; the pain and temperature declaration below the umbilical plane.	Radiculopathy	Myelopathy	Y	Y	Schwannoma	No	Lateral position; Posterior midline approach; Total laminectomy	3h50min	320	GTR

### MRI Characteristics

**Figure 1:**
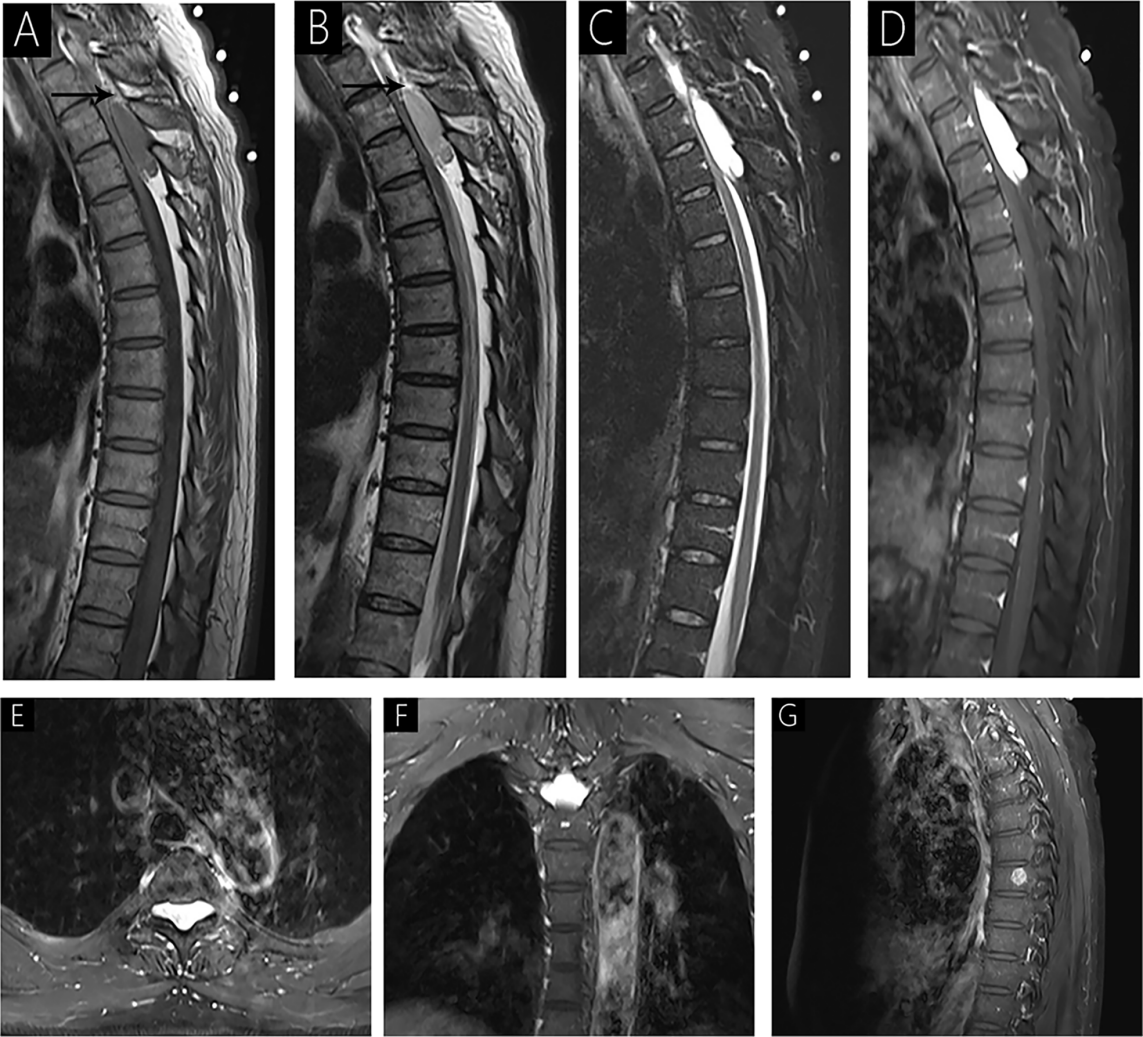
Case 1 (T3-T4) shows an isointense signal on T1WI **(A)**; a mild hyperintense signal on T2WI **(B)**; hyperintense signal on fat-saturated T2WI **(C)**; and homogenous signal with significant enhancement on enhanced MRI **(D–F)**. Sagittal T1WI and T2WI shows an epidural mass causing anterior compression of the thecal sac and peripheral fat tissues (arrow). The spinal cord is markedly compressed with striped slight hyperintensity internally on fat-saturated T2WI **(C)**. Intervertebral foramen extension is noted on axial T1WI with contrast **(E, F)**. A lesion is observed in the 8th vertebra with significant enhancement on sagittal T1WI with contrast **(G)**.

**Figure 2:**
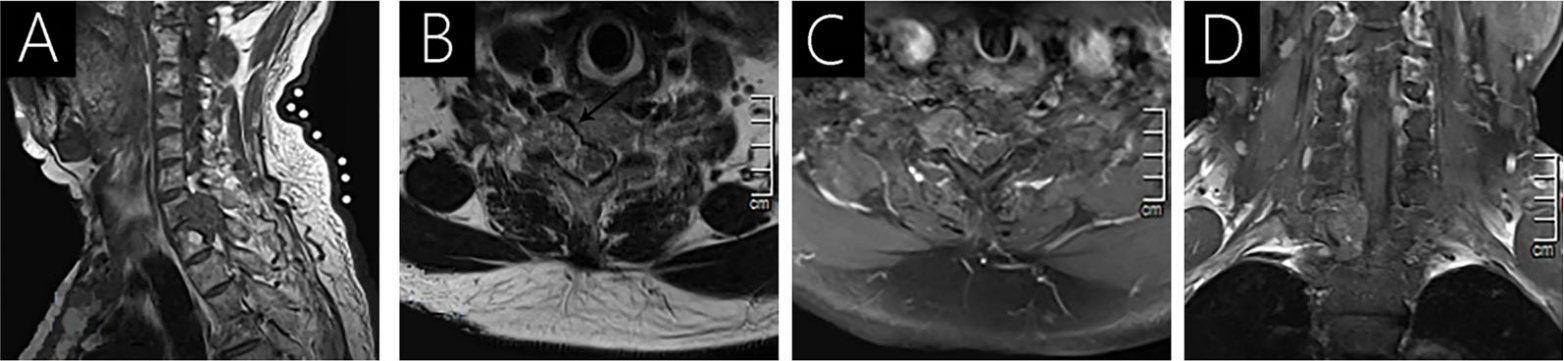
Case 2 (C7-T1) shows an isointense signal on T1WI **(A)**; a heterogeneous intense signal on T2WI **(B)**; and heterogeneous signal with mild enhancement following gadolinium administration **(C, D)**. Vertebral destruction and intervertebral foramen extension are observed on axial T2WI (arrow; **B**) and T1WI with contrast **(C)**.

**Figure 3:**
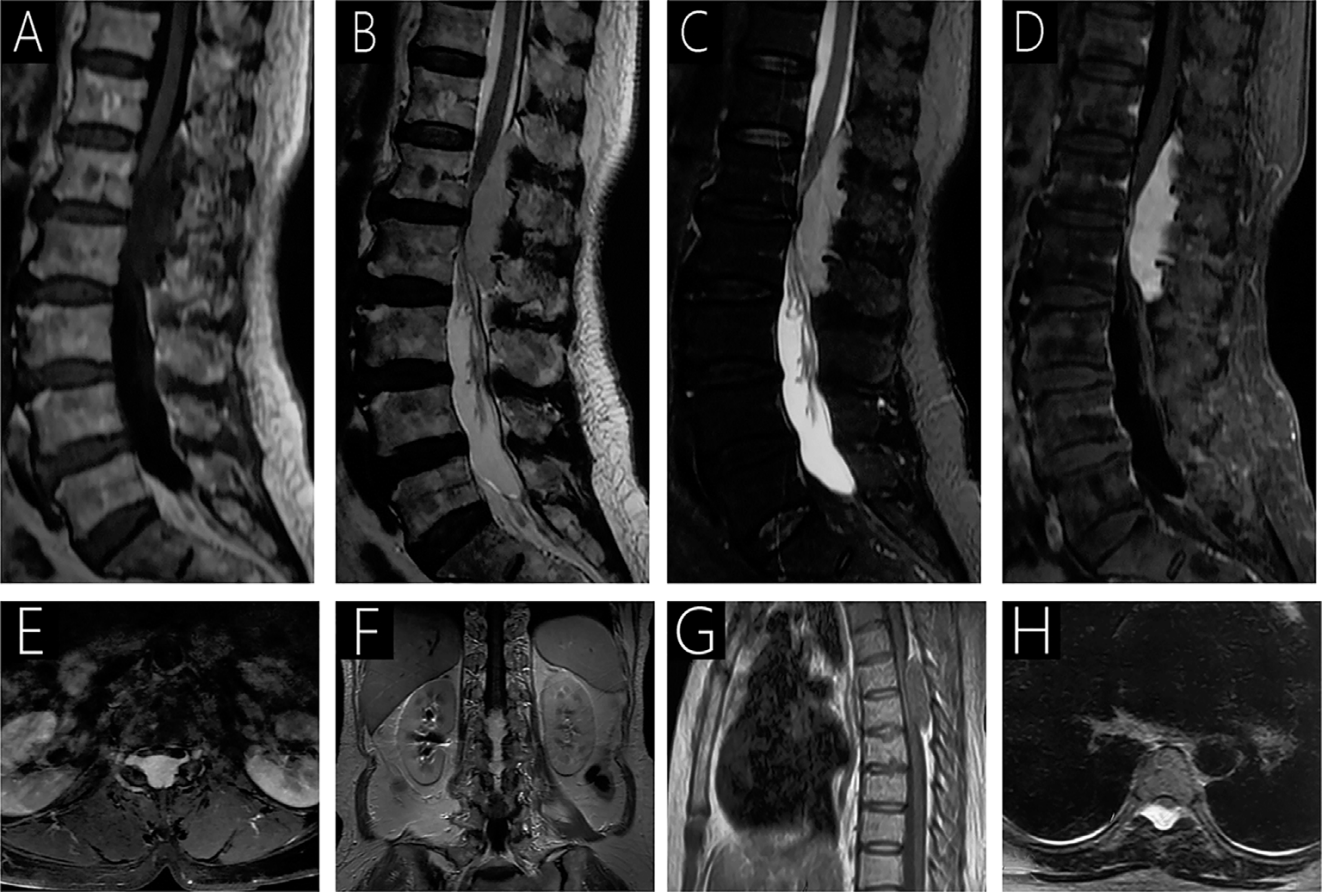
Case 3 (T12-L2) shows a hypointense signal on T1WI **(A)**; a hyperintense signal on T2WI **(B)** and fat-saturated T2WI **(C)**; and heterogeneous signal with mild enhancement following gadolinium administration **(D–F)**. On T2WI, an epidural mass causing anterior compression of the thecal sac and compression of the spinal cord with intramedullary hyperintense signal is observed **(B)**. Intervertebral foramen extension is observed on axial T1WI with contrast **(E)**. Case 4 (T5-T6) sagittal T1WI shows an isointense epidural mass causing anterior compression of the thecal sac **(G)**, and a hyperintense signal is observed on T2WI **(H)**.

**Figure 4:**
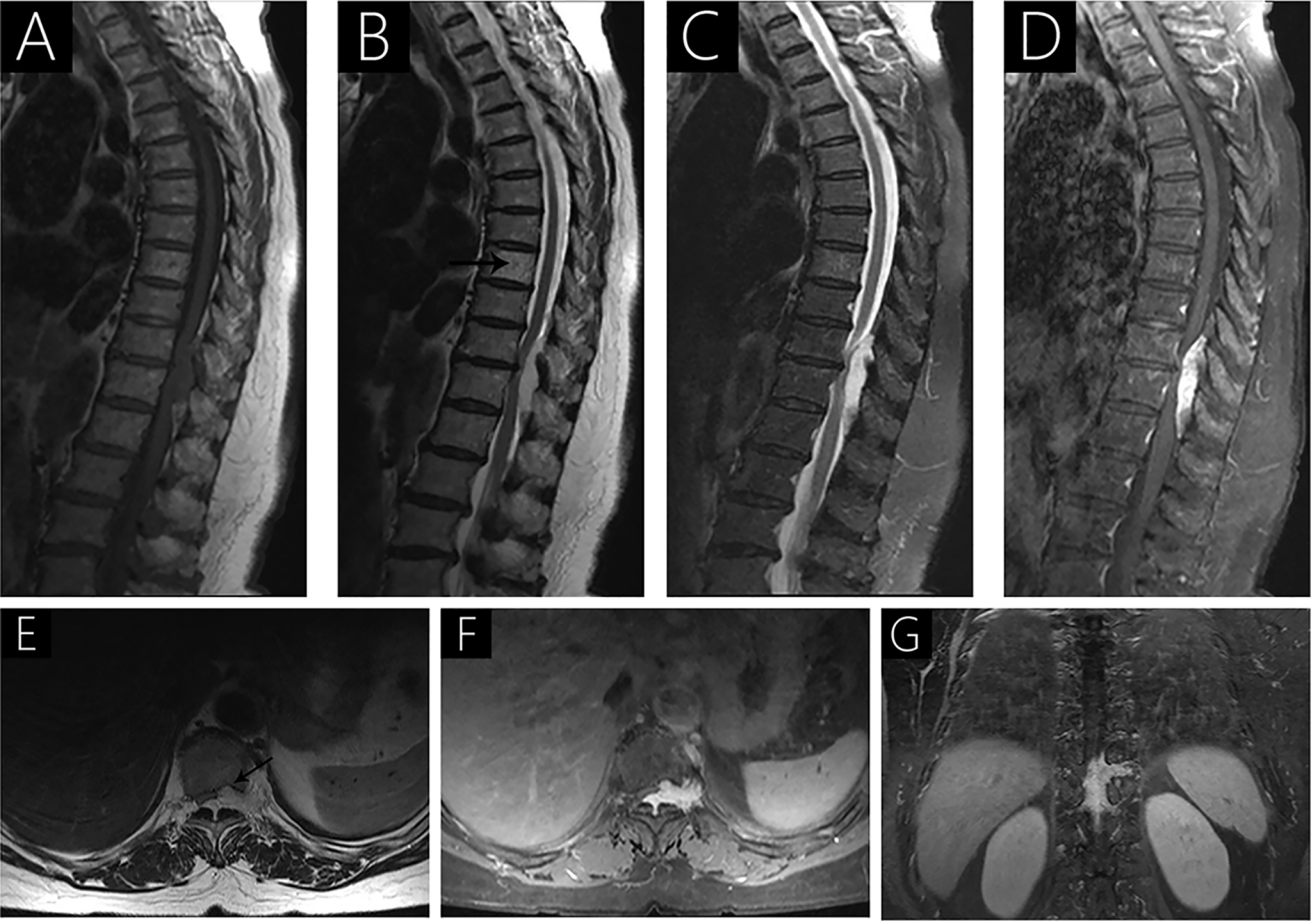
Case 5 (T10-T12) shows a hypointense signal on T1WI **(A)**, a hyperintense signal on T2WI **(B)**, hypo- and hyperintense signal on fat-saturated T2WI **(C)**, and strongly heterogenous enhancement with contrast **(D)** because of intralesional hemorrhage. Sagittal T2WI **(B)** shows a hyperintense signal in the 8th thoracic vertebrae (arrow). Axial T2WI shows vertebral body erosion with well-defined margins (arrow; **E**). Intervertebral foramen extension is observed following gadolinium administration **(F, G)**.

All patients underwent preoperative MRI with and without contrast (**Table 2**, **Figures 1**–**4**). Eight lesions were identified in the dorsal spinal canals (88.9%), and 1 lesion was located on the right of the spinal cord (11.11%; **Figure 2**). In 7 cases, the lesions extended into the intervertebral foramen (77.8%); **Figuress 1E**, **2B**, **3E**, and **4F**), and vertebral destruction was observed in 3 cases (33.3%; arrow marks in **Figures 2B** and **4E**). In 4 cases, abnormal intramedullary signals were observed (44.4%; **Figures 1C** and **3B**). On T1WI, 6 lesions appeared isointense (66.7%) and 3 were hypointense (33.3%). On T2WI, 8 lesions exhibited hyperintense signals (88.9%) and 1 appeared heterogeneously intense (11.1%; **Figure 2B**). On fat-saturated T2WI, 8 lesions showed hyperintense signals and 1 lesion showed hypo- and hyperintense signal because of intralesional hemorrhage (**Figure 4D**). Following gadolinium administration, 5 lesions appeared homogeneous with significant enhancement (55.6%), 3 appeared homogeneous with mild enhancement (33.3%), and 1 appeared heterogeneous with significant enhancement (Case 2; 11.1%). In both Cases 1 and 5, a lesion was detected in the 8^th^ thoracic vertebra, with mild hyperintense signals on T1WI and hyperintense signals on T2WI (arrow; **Figure 4B**). After gadolinium enhancement, the lesion presented significant enhancement in Case 1 (**Figure 1G**); however, Case 5 did not show any enhancement (**Figure 4D**). Imaging diagnosis confirmed vertebral hemangioma in both cases.

**Table 2:** Preoperative MRI of 9 patients with pure spinal epidural cavernous hemangiomas.

Case No.	Spinal level	Location	Intervertebral foramen extension	Vertebral destruction	Intramedullary abnormal signal	Mergering vertebral hemangioma	T1	T2	Fat-saturated	Enhancement
1	T3-T4	Dorsal	Y	None	Y	Y, an abnormal signal was seen in the 8^th^ thoracic vertebra, considering hemangioma.	Isointense	Mild hyperintense	Hyperintense	Homogenous and significant
2	C7-T1	Right	Y	Y	None	None	Isointense	Heterogeneous	Hyperintense	Heterogeneous and mild
3	T12-L2	Dorsal	Y	None	Y	None	Hypointense	Hypointense	Hyperintense	Heterogeneous and mild
4	T5-T6	Dorsal	None	None	None	None	Isointense	Mild hyperintense	Hyperintense	Homogenous and significant
5	T10-T12	Dorsal	Y	Y	None	Y, an abnormal signal was seen in the 8^th^ thoracic vertebra, considering hemangioma.	Hypointense	Hyperintense	Hypo+ Hyperintense	Heterogeneous and significant
6	C6-T1	Dorsal	None	None	None	None	Hypointense	Hyperintense	Hyperintense	Homogenous and significant
7	L4-L5	Dorsal	Y	Y	None	None	Isointense	Mild hyperintense	Mild hyperintense	Heterogeneous and mild
8	T7-T9	Dorsal	Y	None	Y	None	Isointense	Hyperintense	Hyperintense	Homogenous and Significant
9	T6-T9	Dorsal	Y	None	Y	None	Isointense	Mild hyperintense	Hyperintense	Homogenous and mild

### Preoperative diagnosis

The preoperative diagnosis indicated schwannomas in 5 cases (55.6%), ECH in 3 cases (33.3%), and meningioma in 1 case (11.1%).

### Surgical Procedure and Findings

All patients underwent early microsurgery under neuronavigation and electrophysiological monitoring. All microsurgeries were performed in the lateral position *via* the posterior midline approach. Five patients underwent total laminectomy (55.6%), and 4 underwent hemilaminectomy (44.4%). The lesions were always well-circumscribed, reddish, dark reddish, or purplish-red in color, and of a soft consistency. The blood supply was moderate to rich. Anatomically, abnormal proliferation of vascular malformation was usually detected, and prominent small feeding arteries and draining veins were visible. Notably, bleeding on contact with the instrument was a predominant characteristic of lesions. The surface of the lesions was coagulated to cause shrinkage of lesions, which facilitated the careful resection of the lesion. Complete lesion resection was achieved in all cases.

### Histopathological Findings

**Figure 5:**
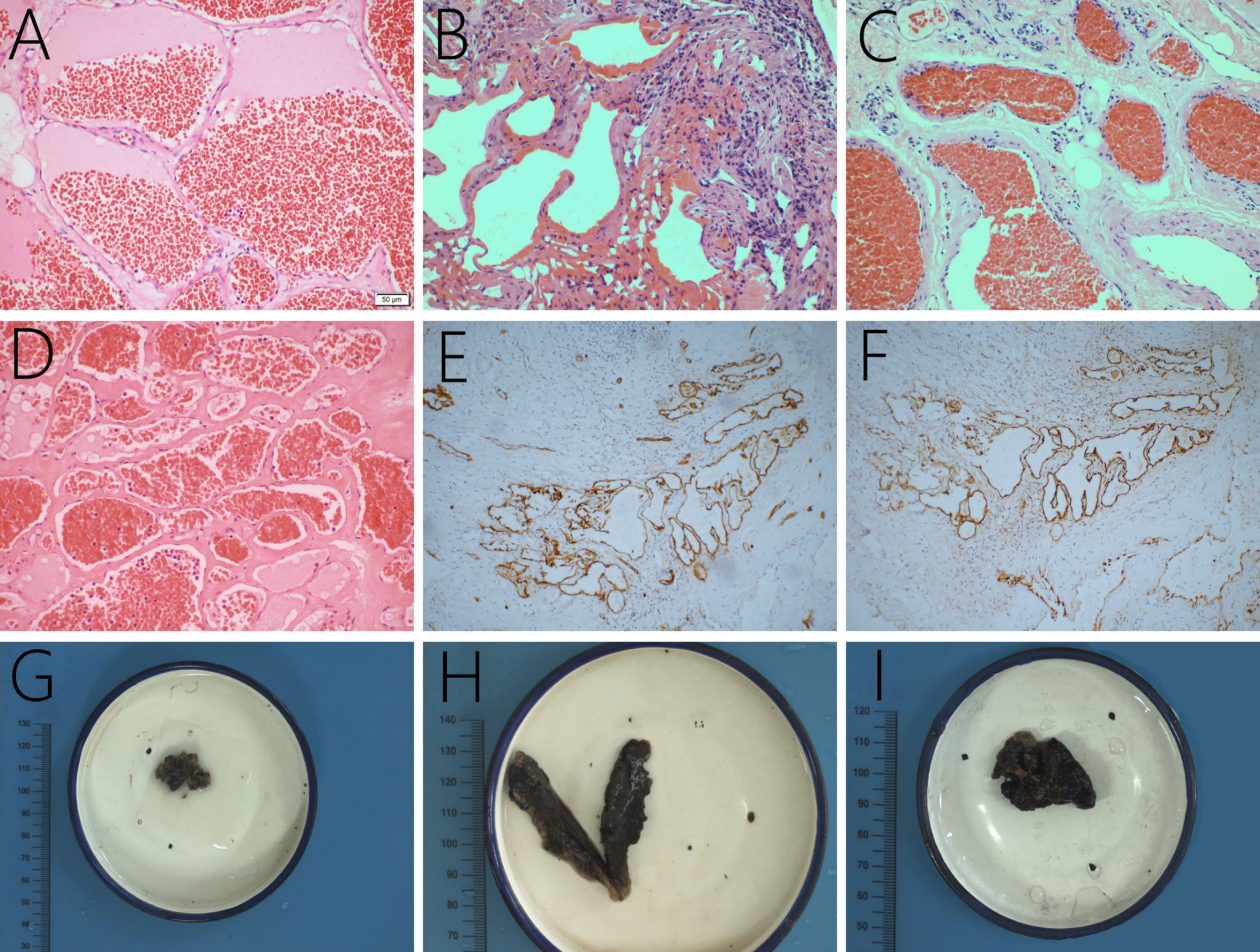
The lesions are mainly composed of thin-walled vascular channels in collagenous connective tissue, lined by a single layer of endothelial cells, and red cells filled in the vessels, original magnification ×200 **(A–D)**. In Case 2, the lesion is found positive for CD34 **(E)** and D2-40 expression **(F)**. Grossly, the lesions are brown and black with a soft texture and a clear boundary (**G–I**, the gross specimens of Case 2, 3, and 7).

In this case series, the lesions comprised of mature thin-walled blood vessels and sinuses were filled with blood and thrombi in varying degrees (**Figure 5**). In Case 2, further immunohistochemical examination revealed positive immunostaining for CD34 (**Figure 5E**) and D2-40 (**Figure 5F**). CD34, as an endothelial cell marker, is usually used in the diagnosis of tumors derived from vascular endothelial cells. D2-40 is expressed on the surface of lymphatic endothelial cells and mesothelial cells to identify whether there are lymphatic vessels in the lesion.

### Follow-Up

Postoperative follow-up was achieved in all 9 patients, with an average follow-up duration of 55.1 months (range, 10–123 months; **Table 3**). Periodical follow-up MRI revealed no recurrence in any patient. Frankel Grade C was observed in 1 case, Grade D in 6 cases, and Grade E in 2 cases, postoperatively. In addition, all cases recovered to Frankel Grade E until the last follow-up in May 2020. Furthermore, limb motor functions returned to normal, resulting in improvements in activities of daily living.

**Table 3:** Preoperative, postoperative function and follow-up with Frankel grade.

Case	Frankel Grade			Follow-up	Outcome	Recurrence
No.	Preoperative	Postoperative	Last follow-up	(months)		
1	D	D	E	10	The double lower limbs myodynamia improved to V Grade postoperatively and the patient leading a normal daily living.	None
2	E	E	E	39	The four limbs’ myodynamia improved to V Grade postoperatively and the patient leading a normal living.	None
3	C	D	E	47	The double lower limbs myodynamia improved to IV Grade postoperatively. The four limbs’ improved to V Grade and the patient leading a normal living.	None
4	D	D	E	41	The four limbs’ myodynamia improved to V Grade and the patient leading a normal living.	None
5	C	D	E	47	The double lower limbs myodynamia improved to IV Grade postoperatively. The four limbs were of V Grade on follow-up and the patient leading a normal living.	None
6	D	D	E	56	The double upper limbs myodynamia were IV Grade postoperatively. The four limbs were of V Grade on follow-up and the patient leading a normal living.	None
7	D	E	E	102	The double lower limbs myodynamia improved to V Grade postoperatively. The four limbs’ were of V Grade on follow-up and the patient leading a normal living.	None
8	B	C	E	123	The double lower limbs myodynamia improved to III Grade postoperatively. The four limbs were of V Grade on follow-up and the patient leading a normal living.	None
9	D	D	E	31	The double lower limbs myodynamia improved to IV Grade postoperatively. The four limbs’ were of V Grade and the patient lead a normal living during the follow-up of 31 months.	None

## Discussion

Spinal ECHs are very rare benign vascular malformations with slow progression. Globus and Doshay first described these lesions in 1929 (14). ECHs can develop anywhere along the spinal canal but predominantly develop at the thoracic levels (15–17), with a predilection for the T2-T6 segment and multisegment involvement (18–20), followed by cervical, lumbar, and sacral levels in the order of reducing occurrence (11). The clinical manifestations of spinal ECHs are primarily dependent on their localization. In our series, the lesions were predominantly localized at the thoracic spine in 5 patients (55.6%), cervicothoracic junction in 2 patients (22.2%), thoracolumbar junction in 1 patient (11.1%), and lumbar spine in 1 patient (11.1%). The ECH lesions are frequently found at the dorsal or dorsolateral region of the spinal canal, wherein the venous plexus is abundant with relatively large accessible spaces (16, 18, 19). Moreover, negative thoracic cavity pressure may facilitate the expansion of the ECH to the pleural cavity (15). In our series, lesions in 8 cases were located dorsally, and that in 1 case was isolated to the right lateral region of the spine. Previous studies have demonstrated a wide variation in patients’ age, ranging from 23 months to 81 years; however, ECHs occur most frequently in adult patients aged between 30 and 60 years (mean age, 47.7 years) with the mean age in men and women reported to be 51 years and 42.6 years, respectively (1, 15, 19, 21, 22). Accumulating studies have also indicated a female predominance in ECHs (9, 16, 17). However, in our series, the mean age was 61 years with a slight male predominance (male:female sex ratio: 5:4), and this discrepancy may be attributed to the small sample size of our study.

### Pathogenesis of ECH

Although the pathogenesis of ECH remains elusive, the development and progression of these entities can be aggravated by the presence of certain predisposing conditions, including pregnancy (2, 7), trauma (18), excessive exercise (16), use of anticoagulants (15), and irradiation (16). In the present study, only Case 8 had a history of falls, and the symptoms gradually intensified within a month. However, in other patients, no such predisposing factors were observed. Thus, various different hypotheses have been proposed to explain the formation of ECHs; however, the specific pathogenesis remains unclear. The blood vessel progenitor dysplasia theory suggested the involvement of progenitor cells in the pathogenesis of these vascular entities (23, 24). Some studies speculated that these lesions originate from the dysplasia vessel-forming mesoderm (23). According to this hypothesis, when the embryonic primordial vessels cannot completely differentiate, ECH may occur (23, 25). Furthermore, the telangiectasia theory assumed that a vascular malformation develops from telangiectasia, which gradually increases in size (26). The third hypothesis is the hereditary theory. The familial occurrences and inheritance studies of these lesions have indicated that ECH might be a hereditary disease with a possible genetic association (23, 27), and some studies have postulated that it is inherited as an autosomal dominant trait with variable expression (10). In our study, we observed two cases of pure ECH combined with vertebral hemangioma, which further support this hypothesis.

In addition, to some extent, pregnancy may explain the female predisposition as pregnancy is considered to be a significant risk factor for the development of neurological signs and symptoms in cases with dormant hemangiomas. Conceivably, gravid uterus impedes the blood flow from the paravertebral veins into the inferior vena cava. The increasing venous pressure can cause the distension of the vascular channels in epidural hemangiomas, leading to a rapid increase in their sizes (3, 7). This is the most critical phenomena contributing to the clinical manifestation of pregnancy-induced symptoms. Furthermore, it can also explain the relatively acute onset of symptoms in the third trimester, as the rapidly enlarging uterus increases the intraabdominal and intrathoracic pressure (28). Symptoms may resolve in the early postpartum period, as a result of the rapid decrease in the pressure caused by the gravid uterus and the correction of the venous blood flow reversal (28). In contrast, the overexpression of angiogenic factors during embryogenesis enhances the occurrence of ECH (29). Estrogen has also been well-recognized to play a crucial role in the development of these lesions by directly acting upon the endothelium of the vascular channels (30, 31). No gestational women were reported to have ECH in our case series; however, ECH during pregnancy has been reported in 8 cases previously (2, 7, 21, 28–32).

### Clinical Characteristics

The clinical presentations are primarily determined by the localization, growth rate, and intra- or extra-lesion hemorrhage of the malformation (10, 33). Generally, ECHs present with chronic progressive myelopathy and/or radiculopathy (1, 10, 11, 17), and the myelopathy seems more frequent than the radiculopathy (15). This might be attributable to a better capacity of nerve roots, contrary to the spinal cord, to tolerate long-term soft compression (2, 15). Myelopathy was observed in 8 cases (except Case 2), and radiculopathy was noted in 3 cases (Case 3, 5, and 9) in our study. Moreover, an acute presentation (11% to 21% of cases) could always be observed presenting with a sudden onset of severe local pain, followed by the rapid development of paralysis, sensory level, and urinary/fecal incontinence (3, 15, 21). The sudden onset of symptoms is always secondary to the significant enlargement of the lesion by intralesional hemorrhage, thrombosis, increased vascularization caused by hormonal effects, or mechanical venous occlusion (18, 23, 34, 35), and the resulting motor deficits are severe (8, 10, 36). Notably, acute hemorrhage often leads to severe cord damage, requiring emergency surgery to decompress and stabilize the spine; nevertheless, in these cases, even complete lesion resection cannot reverse all preoperative myelopathic symptoms, and the prognosis remains poor (4, 15, 19, 37). However, no acute course was found in our study, and early surgery resulted in a significant improvement in all patients. Sphincter dysfunction has also been cited as a late clinical finding in ECHs (9, 38), which was present in 2 cases in our study (Case 1 and 8). Kuytu et al. also presented that in cases with thoracal and lumbar ECHs, a gradual incidence of neurological deficits is more common, whereas in cases with cervical ECHs, a sudden deficit is more frequent (11).

### Radiological Presentation

MRI is an extremely valuable and accurate preoperative investigation tool for assessing ECHs, and helps assess the location and the relationship of the lesion with the surrounding anatomical structures; thus, it is crucial for surgical planning (11, 18). Computed tomography (CT) is also useful for delineating the connections with the surrounding bone or extension into vertebral foramina (1, 18). ECHs always present characteristic lobulated-shaped or spindle-shaped masses filling the epidural space around the spinal cord, with two tapered ends (19, 39). Furthermore, the lesions most often involve multiple vertebral segments, in contrast to arteriovenous malformations, which typically occur at one vertebral segment and usually exhibit a flow void signal on MRI because of high blood velocity (16, 40). In our study, all lesions involved two or more segments. Intervertebral foramen extension is observed frequently and is usually isolated (36), which might be because of the loose tissue structure inside the neural foramina (15, 16, 41); however, pure foraminal and extraforaminal ECH are extremely rare (16). Seven cases presented with intervertebral foramen extension in our study; however, no case of pure foraminal ECH was observed. In addition, pure ECHs rarely cause vertebral destruction (16), which is an occasional observation in cases of recurrence (15). However, in this study, vertebral destruction was observed in 3 cases (Case 2, 5, and 7). In previous studies, homogeneous hypo- to isointensity signals were usually observed in the spinal cord on T1WI with hyperintense signals on T2WI, because of slow blood flow, together with an intense homogeneous signal on contrast enhancement (1, 3, 11, 22, 35). Besides, lesions with hemorrhage, liquefaction of a hematoma, or intravascular thrombosis can always show heterogeneous enhancement (9, 16). In this study, 5 cases exhibited homogeneous enhancement, and 4 cases showed heterogeneous enhancement. For hemorrhagic ECHs, hyperintense signals are seen on both T1WI and T2WI in the acute stage (18, 23). ECHs extending through the intervertebral foramen into the extraspinal region assume an “hourglass” or “dumbbell” shape on axial MRI (**Figure 2C**) (3, 36), and are almost always misdiagnosed as schwannomas, which was the most common preoperative differential diagnosis in this study (5 cases, 55.6%). However, notably, schwannomas exhibit heterogeneous signal intensity, particularly on enhanced MRI (3), and are frequently associated with cystic changes (15). Furthermore, schwannomas always present with an enlarged foramen because of the compression of the adjacent bone (3, 15). Meningioma is another common differential diagnosis, which typically shows as isointense to the cortex on T1WI and T2WI and shows strong homogeneous enhancement with a dura tail sign on post-contrast imaging (16). Peripheral fat tissue is another characteristic finding for differentiating ECHs from the epidural hemorrhagic mass, which is an acute entity and lacks peripheral fat tissue (arrow in **Figures 1A** and **B**) (3). Also, intramedullary hemangiomas always exhibit a mixed signal with a hyperintense hemosiderin ring on T2WI because of repeated bleeding, which is usually not observed in ECHs (16, 17, 19). This might be attributed to the easier elimination of peripheral hemosiderin outside the blood–brain barrier given a richer vascularity of epidural lesions (7, 15, 42).

### Differential Diagnosis

Previous reports have described a wide-ranging differential diagnosis for ECH, including meningiomas, neurinomas, neurofibromas, schwannomas, metastases, angiolipomas, lymphomas, and also disc herniation, synovial cyst, granulomatous infection, pure epidural hematoma, extramedullary hematopoiesis, and spinal canal stenosis (1, 2, 11, 18, 39, 43). In this review, we do not intend to discuss these diagnoses in detail. Lymphoma usually presents with an isointense signal on T2WI and exhibits less frequent paravertebral extension and intervertebral neural foraminal widening (15). Angiolipoma is typically hyperintense on T1WI because of its fat content, whereas the fat in ECH is usually absent and located at the periphery (15, 44). Angiography does not usually detect ECHs (36), and digital subtraction angiography (DSA) has no role in the management of ECHs as these lesions fail to show up on angiography because of their slow blood flow and cannot be embolized (1, 12, 18, 45). However, DSA can be performed in patients with acute symptom onset caused by hemorrhage to rule out other vascular malformations if the patient’s condition allows for imaging and the patient shows nontypical imaging characteristics (21). Arteriovenous malformations always present with flow void signal on MRI because of high-velocity blood flow (15). Notably, an urgent exploratory laminectomy/decompression should be performed on the appearance of acute paraplegia, and DSA should not delay the surgery (33). However, no spinal CT or DSA was performed in our study.

### Histopathological characteristics

Histopathologically, the lesions were comprised of mature thin-walled vessels lined with endothelium and intervening loose connective and adipose tissue (12, 23, 34, 36, 40). The walls of vessels are predominantly lined with single layers of flattened endothelial cells in collagenous tissue without elastic and muscular tissue or neurons (12, 18, 23, 34, 36). Thrombosis and previous residual hemorrhages could also be observed within cavernous hemangioma, particularly in more extensive lesions (18, 30, 37).

### Treatment

#### Surgical Intervention

Early surgical resection with microsurgical technique is considered to be the best and first choice of treatment for ECHs (9, 11, 16, 46, 47), with the aim of total gross resection (17), given that the extent of resection is the most important predictors of recurrence and symptom improvement with a reasonable recovery (1, 18, 35, 48). Besides, en bloc excision may prevent intraoperative bleeding (16). Conservative management is not recommended as ECHs may cause hemorrhage and will not regress with time (22). All our patients provided consent for surgical treatment, and complete resection was achieved in all patients as per postoperative MRI evaluation. Severe intraoperative bleeding and the foraminal, anterior, or intrathoracic extension are major contributing factors limiting a complete resection (15, 17). Notably, even when complete excision is accomplished, symptoms may persist in a few patients, perhaps because of scarring around the dural sac or involvement of nerve roots, and does not necessarily indicate lesion recurrence (3). Moreover, most patients are surgically cured and show complete recovery of neurological symptoms with a good prognosis (11, 15). In this study, all patients exhibited significant improvement with a favorable outcome; however, occasional numbness around the incision was also noted. The lateral position during surgery has been recommended in some reports to clear the operating field, control intraoperative bleeding, and reduce epidural venous pressure resulting from the reduced chest and abdominal compression in the vertebral canal (15). The lateral position, combined with a posterior midline approach, was selected for all the cases in our study. Progressive shrinkage of the volume of hemangioma with bipolar coagulation of its surface can effectively reduce the unpredictable incidence of intraoperative bleeding (35) and facilitate the separation of the lesion (15). Previous clinical reports have also recommended approaching from the region not in contact with the thecal sac (1, 36, 48). Moreover, a preoperative diagnosis of ECH was highly significant, as these vascular malformations carry the risk of massive bleeding, as shown in our study, and may not be amenable to total resection without thorough preoperative assessment and surgical planning.

#### Radiotherapy

For residual lesions, adjuvant radiotherapy is not recommended considering the potential for radiation damage to the spinal cord (3, 16). However, radiotherapy has also been sporadically reported to be beneficial in patients with incomplete resection (3, 13, 18, 35). Furthermore, Sohn et al. (13) reported a case with stereotactic radiotherapy (32 Gy in 4 fractions). No radiotherapy was conducted in our case series, and for residual ECHs, we recommend a close follow-up with MRI monitoring and avoiding a second surgical resection until the lesion size allows for one (3, 47).

### Prognosis

The prognosis of patients with ECH is generally good, with a recovery rate of up to 92% (48). In our study, the median follow-up duration was 47 months, with seven of the nine cases having a follow-up period of more than 3 years, and no recurrence was observed during follow-up MRI. All patients experienced gradual neurological improvement and a good outcome. These results indicated that surgical intervention was effective (9, 16). Notably, the severity of preoperative neurologic condition appears to be the most significant prognostic factor (1), and severe cord damage resulting from acute hemorrhage may correlate with a poor functional prognosis or adversely influence the outcome (1, 15). Therefore, early surgical treatment is recommended to prevent irreversible neurological damage (17). However, it is also worthwhile to further evaluate surgical opportunities in asymptomatic patients with large epidural cavernous hemangiomas (15). Thus far, the potential for malignant transformation of ECHs has not been reported in the literature (48).

## Conclusion

Pure ECH is a rare vascular malformation in the spine, and early surgical resection is the best choice of treatment with a good prognosis; however, for ECH with acute hemorrhage, complete recovery cannot always be achieved even with emergency decompression and complete resection. More reported cases with long-term follow-up are needed to completely understand the etiopathogenesis of pure ECH. Our study might represent an additional reference among the few available case series that might serve as a prospective guide for clinicians and radiologists.

## Data Availability Statement

The original contributions presented in the study are included in the article/supplementary material. Further inquiries can be directed to the corresponding author.

## Ethics Statement

The studies involving human participants were reviewed and approved by Ethics Committee of the First Hospital of Jilin University. The patients/participants provided their written informed consent to participate in this study. Written informed consent was obtained from the individual(s) for the publication of any potentially identifiable images or data included in this article.

## Author Contributions

LZ and YJ made study design, data collection, data analysis and interpretation, and composed the manuscript and literature review. YL and YW were the surgeon that performed the surgery and did data collection, data analysis, and interpretation. YS and YB made English and grammar corrections, critical revisions, and approved final version. YL had the acquisition, analysis or interpretation of data for the work, revising it critically for important intellectual content, final approval of the version to be published, and agreement to be accountable for all aspects of the work in ensuring that questions related to the accuracy or integrity of any part of the work are appropriately investigated and resolved. All authors agree to be accountable for the content of the work. All authors contributed to the article and approved the submitted version.

## Conflict of Interest

The authors declare that the research was conducted in the absence of any commercial or financial relationships that could be construed as a potential conflict of interest.

## Abbreviations

ECH, epidural cavernous hemangioma; CNS, central nervous system; MRI, magnetic resonance imaging; CT, computed tomography; DSA, digital subtraction angiography; T1WI, T1-weighted imaging; T2WI, T2-weighted imaging; GTR, gross total resection; STR, subtotal resection.
